# Estimating the number of HIV infections averted: an approach and its issues

**DOI:** 10.1136/sti.2008.030247

**Published:** 2008-07-22

**Authors:** L M Heaton, R Komatsu, D Low-Beer, T B Fowler, P O Way

**Affiliations:** 1 US Census Bureau, Washington, USA; 2 The Global Fund To Fight AIDS, Tuberculosis and Malaria, Geneva, Switzerland; 3 Cambridge University Health, Judge Business School, Cambridge University, Cambridge, UK

## Abstract

**Objective:**

To propose a methodology to estimate the number of new HIV infections averted. Knowledge of HIV infection has increased tremendously and modelling tools to project current epidemics into the future have greatly improved. Different types of models can be used to estimate HIV infections averted, although the number of new HIV infections averted cannot be measured directly.

**Method:**

Using cohort-component population projections, a disease modelling-based approach was used to compare the observed epidemiology of a disease after programme initiation with an expected epidemiology from past trends before programme initiation. The concept of modelling infections averted in a disease modelling-based approach involves a comparison between an “expected” or baseline epidemic with an “estimated” one. A hypothetical example was featured in order to demonstrate the proposed methodology. Using both the Estimation and Projection Package (EPP) and the Spectrum demographic modelling program, the underlying annual incidence levels implied by both the baseline and estimated epidemics were examined.

**Results:**

The difference between baseline and estimated incidence levels is interpreted as “infections averted”. Strengths and limitations of the approach are discussed.

**Conclusions:**

In this study an expected epidemiological approach was compared to one based on observation. Once sufficient data become available, the validation of various country data including HIV prevalence, mortality, and behaviour must be done. Additional information related to behaviour change may be critical to further support arguments for a change in disease trend. It is therefore important to use all available data, consequently strengthening findings from a disease modelling-based approach on HIV infections averted.

At present, the number of people living with HIV and AIDS continues to increase in many areas of the world. At the same time, we have the HIV prevention and treatment strategies and tools to mitigate the epidemic. To meet the challenges ahead we must continue to strengthen these. Effective prevention and treatment activities go towards averting potential HIV infections that would otherwise occur. Voluntary counselling and testing, condom social marketing, antiretroviral use and mass media campaigns are only a few of many strategies employed to curb HIV transmission.

In order to gauge the possible impacts of prevention and treatment programmes, a methodology must be in place to estimate HIV infections averted. In this paper, we introduce three basic approaches to estimate HIV infections averted. One of the three approaches, a disease modelling-based approach, is discussed at length.

## Coverage-based approach

In this approach, estimation of new infections averted involves estimating the possible outcome of a proposed intervention. This estimate is typically based on a literature review and an assumption of the intervention’s coverage. Evaluating the impact of a health intervention on disease is of great interest in the field of public health. One reason for this interest is to advocate for certain intervention programmes to reduce or halt the spread of a disease. Estimating the number of infections averted attributable to such programmes can also provide impetus to establishing the program, help focus programme activities, and set goals.^1^^–^9 Determining how much to scale up programmes to reach a larger number of people in need of the intervention is also of concern.^10^^–^12 An analysis of this approach is hypothetical in that we are answering questions such as “If male circumcision rates increase by 100% over a specific period of time, what are the possible number of HIV infections that might be averted over that time period?” Thus, the coverage-based approach can be valuable for public health advocacy purposes.

## Behaviour-based approach

HIV infections are mediated by behaviour with interventions intended to change risky behaviours. A model can be constructed to evaluate these interventions for their effectiveness. This is known as a “behaviour-based approach”, as HIV infections are mediated by behaviour with interventions aimed at changing risky behaviours.^13^^–^15 Most available interventions intend to reduce risky behaviours, thereby reducing new infections. Thus, if the effect of a behaviour change on new infections and the prevalence of changed behaviour are known (or assumed), mathematical models can be constructed based on behaviour. However, the relation between behaviour and disease is complex and dependent on the epidemiological context.^15^ ^16^ Cost-effectiveness analysis of intervention programmes often occurs in both the coverage-based and behaviour-based approaches when selecting or advocating for a certain intervention.^4^ ^7^^–^9 11 13 14 A major problem with the behaviour-based approach is that there is a clear lack of relevant behavioural data to use in many developing countries, although there have been an increasing number of behavioural surveys. Therefore, the behaviour-based approach is difficult to implement currently in any reliable way in most developing countries.

## Disease modelling-based approach

In this approach, the observed epidemiology of a disease after intervention can be compared with its past epidemiology or an expected epidemiology based on the past trend. This approach has been used to demonstrate the effect of a vaccination programme by showing a secular trend before and after introduction of the programme.^17^

In all three approaches, coverage, disease and behaviour, the number of infections averted needs to be estimated through mathematical modelling since it cannot be measured directly (that is, by definition, it is a non-event). Many studies in the coverage-based and behaviour-based approaches focus on the hypothetical analysis of the effect of certain interventions on disease trends. However, reliance on actual epidemiological change and an attempt to directly estimate infections averted based on the actual epidemiology makes the disease modelling-based approach preferable. A disease modelling-based approach for HIV infections averted is inherently theoretical, unlike control of endemic disease through biomedical solutions such as immunisation. It is of special importance, therefore, to validate and alter baseline curves based on the best available data in a disease modelling-based approach.

In this paper, we focus on the disease modelling-based approach to estimating new HIV infections averted. In brief, baseline projections of HIV incidence are prepared for countries using data pertaining to the time period before intervention programmes were instituted. This baseline will serve as a reference for future comparisons. In this paper, we use data for years through 2004 to derive the baseline projection.^18^ Subsequently, the incidence trend will be re-estimated for countries using additional epidemiological surveillance data available for years post-2004. The re-estimated incidence trend, compared to the baseline trend, will represent the change in the epidemic that may be the result of a number of factors including programme changes. The difference in the number of new HIV infections implied by the prevalence levels will be taken as the number of infections averted.

We examined several countries with sentinel surveillance data for years 2005 and 2006 and found that in the case of one of those countries, only a few thousand HIV infections were averted by 2010. In another country, no new HIV infections were averted. Still, in another country, there were more infections in the re-estimated trend than there were in the baseline projection. To properly estimate infections averted, we need a sufficient number of years’ worth of data post-2004 to gauge impacts. The major obstacle in carrying out this methodology with country-specific data is that insufficient sentinel surveillance data are available beyond 2004 to make this feasible at present. Validation of the results of the methodology proposed in this paper must await further data. Therefore, we demonstrate this model by illustrating a hypothetical example. Applications of such a modelling approach are discussed along with issues relevant to using this approach.

## METHODOLOGY

The modelling process begins with undertaking an extensive review of data that contain information on epidemiology, population, fertility, mortality and migration. Sources include census and survey data, refugee and labour migration statistics, vital registration statistics, administrative records and HIV surveillance data. Once this review is complete, the new information is reconciled with the historical information. A decision is then made on whether a revision of previous population estimates and projections is warranted.

Although it has been clear for a number of years that mortality estimates and projections for many countries would have to be revised because of AIDS mortality, the lack of accurate empirical data on AIDS deaths, the paucity of data on HIV infection among the general population and the absence of tools to project the impact of AIDS epidemics into the future have all hampered these efforts. Although the accuracy of data on AIDS cases and AIDS deaths has not substantially improved, our knowledge of HIV infection has increased tremendously and modelling tools to project current epidemics into the future have greatly improved.

### Development of adult HIV prevalence estimates

The Estimation and Projection Package (EPP) computer software is used to estimate and project adult (15–49) HIV prevalence from sentinel surveillance data in countries with generalised epidemics, defined as an epidemic with national-level prevalence above 1%.^19^^–^21

In countries with a generalised epidemic, national estimates of HIV prevalence are typically based on data generated by surveillance systems that focus on pregnant women who attend a sample of sentinel antenatal clinics. Typically, an urban and a rural epidemic are derived, as the epidemics are normally distinct in most settings. The EPP software will then aggregate these two sub-epidemics to the national level. The national level results are used to obtain HIV prevalence and incidence curves used in estimation of HIV infections averted.

This method assumes that prevalence among pregnant women is a good approximation of prevalence among the adult population aged 15-49 in countries with a generalised HIV epidemic.^22^ National population-based studies that include HIV testing have been completed in several countries in recent years.^23^^–^25 Comparison of these results with those from the antenatal clinic data has provided evidence that the use of antenatal clinic data to model the epidemic among the adult population is appropriate.^22^ ^23^ ^26^^–^28 There are some recent examples of considerably lower national HIV prevalence from population-based surveys, such as in Kenya. Results from these population-based surveys can be used in the EPP software to calibrate the urban and rural curves of HIV prevalence based on antenatal data to a nationally representative total prevalence level.^20^

### Development of the baseline projection

Several stages are involved in the development of the baseline projection. First, hypothetical demographic estimates are prepared that do not include AIDS mortality. The impact of the HIV epidemic is then modelled. Estimates of AIDS mortality are combined with the estimates of non-AIDS mortality to obtain the baseline demographic estimates.

Hypothetical demographic results that do not include AIDS-related mortality are produced using the Spectrum modelling program (The Futures Institute, Glastonbury, CT, USA).

The hypothetical series shows what would have happened if a country had not been affected by the HIV/AIDS epidemic. This modelling takes into account not only lower death rates but also associated changes to a country’s age-sex structure and, indirectly, the combined effects of lower mortality and changing population composition on demographic indicators. This series assumes the same fertility rates (based on observed data) as the series that incorporates HIV/AIDS. The non-AIDS results are then input into the AIDS impact module (AIM) of Spectrum along with the trend in HIV prevalence from EPP and other epidemiological parameters. The estimates of incidence needed to estimate infections averted is one of many outputs from the Spectrum modelling program.

For ease of use, we use the 2006 revision of the *World Population Prospects* from the United Nations Population Division available in Spectrum for the demographic inputs.^29^

Once the AIM module is run, the user has the incidence and prevalence numbers needed for the baseline projection. Many of the inputs used in a typical modelling exercise are those that are based on exhaustive research by the UNAIDS modelling group.^19^ ^20^ ^22^ ^30^ ^31^ This group updates its assumptions when additional empirical research becomes available.

## DEVELOPMENT OF THE NUMBER OF INFECTIONS AVERTED

### Hypothetical example

The concept of modelling infections averted in a disease modelling-based approach involves a comparison between an “expected” or baseline epidemic with an “estimated” one. We examine the underlying annual incidence levels implied by both the expected and estimated epidemics. The difference in the incidence levels is interpreted as infections averted. We use the EPP and Spectrum models to compare fits of the data before any major HIV programme initiatives, the baseline epidemic, and after these initiatives have started, the estimated epidemic. Major programme initiatives by various donors began in 2004. As HIV prevalence data become available for the post-2004 period it is necessary to repeat the steps outlined above for the baseline estimates to incorporate the new data. This will result in new estimates of the number of new HIV cases for each year from 2005 to 2010. The difference between the baseline estimates of incidence and the post-2004 estimates is assumed to represent the number of infections averted during the period of interest.

We model two scenarios—a baseline projection and an assumed 25% decline in HIV adult prevalence from the 2004 level by 2010.

**Figure 1:**
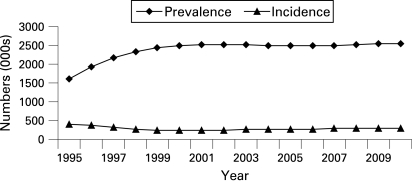
HIV prevalence and incidence baseline scenario: 1995–2010.

In the hypothetical example of modelling infections averted, both the number of baseline HIV infections and the resulting incidence numbers are shown for 1995 to 2010 (fig 1).

**Figure 2:**
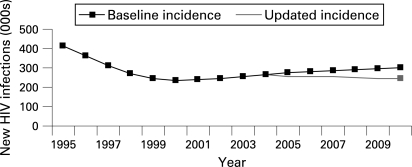
HIV incidence—two scenarios 1995–2010.

We are primarily interested in infections averted after the introduction of prevention programmes, 2005 in this example (fig 2).

**Figure 3:**
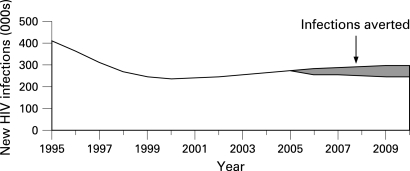
Cumulative number of HIV infections averted: 2005–10.

An estimate of infections averted after 2004, if we assume a 25% decline in HIV prevalence by 2010, would be represented in the shaded area (fig 3). The cumulative total of the shaded area represents the number of new infections that were averted during the period from 2005 to 2010.

## DISCUSSION

The number of infections averted cannot be measured directly, but a disease modelling-based approach is a direct estimation based on actual data from disease surveillance systems. In contrast, a coverage-based approach is indirect estimation based on intervention coverage (hypothetical or measured) and its presumed or estimated effect on disease. Similarly, a behaviour-based approach is indirect estimation based on a change in prevalence of certain behaviours and the estimated effect on a disease.

No approach can be exact since infections averted cannot be directly measured and any models are based on a number of assumptions. None the less, we propose a disease modelling-based approach in this paper to estimate infections averted because this approach is a more direct one. In addition, while we have described estimation of HIV infections averted for countries with generalised epidemics, this methodology can also be used to estimate infections averted for countries with concentrated epidemics as long as there are data to do so.

One use of estimating infections averted is to track progress made by international initiatives. The US President’s Emergency Plan for AIDS Relief (PEPFAR) has a global goal of seven million HIV infections averted between the years 2005 and 2010, and a large amount of financial resources are disbursed in PEPFAR’s 15 focus countries. Another global initiative, the Global Fund To Fight AIDS, Tuberculosis and Malaria, also has a large investment in HIV/AIDS programmes. In general, the impact of donor initiatives cannot be separated in a country. Indeed, prevention requires behavioural change along with enabling factors such as community activities, communication, commodity availability and a favourable legal and environmental framework.^32^^–^42 In the complex environment in which HIV prevention and treatment take place, it would be very difficult to assess a particular programme’s impact.

Several issues related to the approach described in this paper need to be addressed. The first is related to the time lag in the processing and release of surveillance results. It will not be possible to make an estimate of infections averted until well after the post-2004 data are collected. We have observed this when applying the methodology to specific countries. In addition, the approach as described here can result in different historical levels of prevalence and incidence between the baseline scenario and the later scenario based on new HIV prevalence data that will be used to derive infections averted—that is, when the new HIV prevalence data post-2004 are added to EPP, the software will often produce a somewhat different “best fit” of all the data, including the pre-2004 period. To address this issue, the EPP infections-averted fitting process can be adjusted so that the results covering the time period before the infections averted period approximate the original baseline results. This requires adjustment of any of the EPP fitting parameters—or some sort of ratio adjustment. Another way of addressing this issue is to limit new data entered into EPP to only the surveillance sites available at the time the baseline estimate was prepared (for example, only those sites available through 2003 or 2004). However, ignoring data from new sites added to a country’s surveillance system at a later point in time may not be advisable. It will be important to validate the curves to HIV prevalence, mortality, and behavioural data from several sources once sufficient trend data are available.

Another concern is that of antiretroviral use. With widespread use of these drugs in resource-constrained settings, we could see an increase in prevalence, rather than a decline, that is not related to an increase in incidence.^43^ Although it appears that transmissibility of HIV may be reduced among people on antiretroviral treatment, this may be offset by increases in risk-taking behaviour. For example, if people are feeling better as a result of antiretroviral treatment, they may engage in more sexual activity. However, studies have not been conclusive. Even in industrialised settings, there is no strong evidence that such disinhibition occurs.^44^ Therefore, the increase in prevalence due to increased survivorship of those on treatment needs to be factored out of the infections-averted estimation process. The next update of the EPP software will take antiretroviral use into account in order to better fit the models.^45^

Finally, in recent years, a number of countries have carried out population-based surveys that include HIV testing and many more are planned for the future. These surveys are finding different levels of HIV prevalence in the general population from that indicated by antenatal clinic sentinel surveillance. EPP allows for the national HIV prevalence curves to be calibrated to match the results of a population-based survey. At what point do results represent change due to programmes and not just a calibration to the time series? HIV prevalence itself has a range of uncertainty and this may also need to be addressed in infections averted.^46^^–^48

In a stable, endemic situation, a coverage-based approach may well produce convincing models as long as good knowledge of the relation between coverage and disease prevention exists. This is true for a measles vaccine, for instance. However, HIV epidemics are heterogeneous and the relation between coverage and disease prevention may not be the same in all cultural and epidemiological situations.^49^ Even with the behaviour-based approach, knowledge about behaviour change and infections averted is incomplete. Compared to stable endemic diseases, estimating the natural history of an increasing or decreasing epidemic is less straightforward, especially with limited surveillance data.

Infections averted are probably different for different levels of prevalence, but this often cannot be addressed or is only addressed in broad ways such as by differentiating by world regions in either a coverage-based or behaviour-based model. In stable, endemic situations, the disease modelling-based approach is simple and convincing, even without a good understanding of the intervention’s impact on disease and the overall impact on the population, as long as the disease level clearly declines in the post-intervention period compared to an expected level. However, the HIV epidemic continues to evolve. While a coverage-based approach may be a better tool for advocacy and planning for an intervention programme, the disease modelling-based approach is better suited to track the possible effect of the intervention’s actual implementation.

Is integration of all three approaches a solution? Most probably it is. However, implementing integration is very difficult. The reality is far from the ideal owing to various gaps in data and limited scientific understanding and knowledge on the complicated and delicate relations between coverage, behaviour and disease. The Asian epidemic model (AEM) deals with both disease-related and behaviour-related information.^50^ ^51^ It can produce better fitting models to the disease as well as the underlying behaviour and, consequently, scientifically convincing curves. Therefore, infections averted estimated by comparing two scenarios in the AEM application appear quite persuasive. However, data requirements are intensive and can be met successfully by only a few countries—for instance, Thailand. More extensive data collection should become routine as recommended by the World Health Organization and UNAIDS.^52^ ^53^ Periodic analysis of collected data for strategic information purposes facilitates informed decision-making for programme management as well as evidence-based advocacy.

In conclusion, different types of models can be used to estimate HIV infections averted, but no model is perfect. We have delineated one approach here based on an expected epidemiology and an observed one, a conceptually simple approach, but some details still need careful consideration and further research. Moreover, it is important to improve the breadth and the availability of country level data on various aspects of the HIV epidemic and refine the models accordingly. The critical stage is to validate the curves to different country data sources including HIV prevalence, mortality and behaviour. Combining a coverage-based approach with a disease modelling-based approach is not simple because the two approaches have different sets of assumptions. Additional information related to behaviour change may be critical to have in some situations to further support arguments for a change in disease trend.^54^^–^64

**Key messages**

- A disease modelling-based methodology has been proposed to estimate the number of HIV infections averted. It entails examining the number of new incident cases in an expected epidemic based on data from years before enactment of major initiatives to the number of new incident cases in an actual epidemic using data after programme initiatives began.
- It is not possible to attribute the effects of the overall change in incident infections to specific programmes. Rather, an epidemic’s trajectory can be analysed within the specific country context in relation to its overall programme activities and any behaviour change.
- Validation of results using empirical data from different countries needs to be done once sufficient data become available to do so.
